# The impact of university freshmen’s mental health on academic performance: an empirical study based on M University in Fujian Province, China

**DOI:** 10.3389/fpubh.2024.1483031

**Published:** 2024-12-13

**Authors:** Xue Deng, Cheng Li

**Affiliations:** School of Sociology and Political Science, Anhui University, Hefei, Anhui, China

**Keywords:** university freshmen, academic performance, regression analysis, mental health, gender differences

## Abstract

**Objective:**

To investigate the impact of freshmen’s mental health on their short-and long-term academic performance, as well as to provide empirical evidence for improving university students’ mental health intervention tactics and higher education quality.

**Methods:**

A multiple regression model was used to analyze student data from 3 years of enrollment at M University in Fujian Province.

**Results:**

Different mental health problems have a significant impact on academic performance, which varies by gender, enrollment year, and subject. Somatization, anxiety, and depression have a significant negative impact on both comprehensive and single-subject scores, while anxiety, social aggression, and other variables can increase academic performance in certain settings. Some effects are notably moderated by gender and enrollment year.

**Conclusion:**

University officials should strengthen mental health surveillance and intervention during the first few years of student enrollment to mitigate the harmful impact of mental health issues on academic performance. The moderate to strong effect sizes for variables like somatization, depression, and anxiety indicate that early interventions could be crucial in reducing their negative impact on both short-and long-term academic outcomes. Furthermore, the study discovered disparities in mental health and academic performance across students of different genders and enrollment years, emphasizing that educational personnel should design more tailored mental health support methods that consider these differences.

## 1 Introduction

The relationship between mental health and academic achievement has been the subject of educational and psychological research, particularly among university freshmen making the transition to higher education. Because of changes in role adaptation, interpersonal relationships, learning, and lifestyle, university freshmen frequently encounter a number of mental health problems such as depression, anxiety, and stress (1–3). The World Mental Health International College Student (WMH-ICS) survey conducted by the World Health Organization (WHO) shows that approximately one-third of university freshmen exhibit symptoms of anxiety, depression, and other mental health issues (4). Similarly, the study about Chinese university students (5) has demonstrated an upward trend in mental health problems among freshmen, showing that this problem is worldwide and permanent.

University students’ mental health problems have a considerable impact on their academic performance, in addition to their physical health (6–8). Internalizing problems (such as depression, anxiety, and sleep problems) and externalizing problems (such as attention deficiency, hyperactivity, impulsivity, and behavioral disorders) can all hinder a student’s academic performance (9). Studies have demonstrated that mental health disorders severely affect students’ academic performance (10) through many pathways, including attention, motivation, and overall cognitive function (11). While these studies show links between mental health and academic performance, there is still a dearth of in-depth analysis of how numerous mental health disorders interact and collectively affect academic performance.

Many variables contribute to freshmen’s psychological problems, but academic pressure, economic challenges, and social isolation are the primary contributors (12). Mental health problems prior to university enrollment, as well as separation from the original family, can exacerbate the onset of mental symptoms (13). The importance of the original family for mental health should not be overlooked. Original family situations such as family economic status, domestic violence and conflict, and parental infidelity can exacerbate mental health problems for students (14). However, present research usually focuses on the effects of single elements, with no complete examination of how these factors interact and which mediating mechanisms influence students’ mental health and academic performance.

Despite substantial studies on the association between mental health and academic performance, the majority of studies have been dominated by cross-sectional data, limiting comprehension of the long-term impacts of mental health disorders. Furthermore, the majority of this research concentrated on specific mental health aspects and did not provide a comprehensive study of overall mental health. As a result, the purpose of this study is to investigate the short- and long-term effects of university freshmen’s mental health conditions, as well as their various determinants, on academic performance using longitudinal data analysis. With this method, the study seeks to provide a more thorough knowledge of how mental health problems affect academic performance over time, as well as helpful insights for educational institutions in developing effective student mental health support networks.

## 2 Data and method

### 2.1 Data source and evaluation method

The research subjects for this study were students enrolled at M University in Fujian Province in 2016, 2017, and 2018. M University is a full-time public undergraduate institution with approximately 20,000 current students, and its student group can be considered representative of university students in southeast China to some extent. The university students were chosen as the research object because of the ongoing systematic mental health census and its diverse subjects that can provide extensive data support.

The large-scale mental health census for freshmen in October provides data on mental health. The census aims to completely examine students’ mental health, and the data includes several variables such as anxiety, depression, and stress. Academic performance is evaluated using students’ test scores in mathematics (including advanced mathematics and calculus), physics, chemistry, and English, which have become major markers of academic performance due to their fundamentality and universality. The test scores are exported from the educational administration system. To ensure the data’s integrity and accuracy, we removed samples from the four cases of examination absence, delay, pending examination, and violation of discipline. Matching mental census data with academic performance through student ID resulted in a final valid sample of 9,189 students: 3830 enrolled in 2016, 3,548 in 2017, and 1811 in 2018. Not only is the sample size appropriate for statistical analysis, but its time span (three enrollment years) allows for the examination of long-term trends in the impact of mental health on academic performance. The demographic characteristics of the sample are shown in Table 1.

**Table 1:** Demographic characteristics of the sample.

Enrollment year	Gender		
	Male	Female	Both genders
2016	1,628	2,202	3,830
2017	1,626	1922	3,548
2018	690	1,121	1811
Total	3,944	5,245	9,189

This study employed the Chinese College Students Mental Health Scale (CCSMHS) (15), which was designed by China’s Ministry of Education, to assess the mental health of Chinese university students. CCSMHS, which is based on a wide variety of clinical psychology theory and empirical research, is extensively used at many universities in China due to its authority and applicability. It includes 12 characteristics to thoroughly measure the mental health state of university students: (1) Somatization, to determine the psychological reasons for physical discomfort symptoms^1^. (2) Anxiety, to measure the persistent tension and worry; (3) Depression, to determine the extent of continuous low mood and loss of interest in life; (4) Inferiority complex, to assess the individual’s underestimation of their own skill and value; (5) Social withdrawal: to evaluate people’s withdrawal behavior in interpersonal interactions. (6) Social aggression: to assess individual hostility and aggressive behavior during social engagement; (7) Paranoid: to evaluate the inclination to distrust individuals and show excessive suspicion; (8) Compulsion: to evaluate repeating compulsive thinking and behavior; (9) Dependency: to assess students’ feelings of excessive dependency on others. (10) Impulsivity: to measure impulsive acts that are difficult to moderate. (11) Psychosexual disorder: to quantify sexual-related psychiatric distress; (12) Psychotic tendencies: to assess the current tendency of psychotic symptoms.

The scale uses a Likert level 5 scoring approach in which students score the frequency of symptoms reported in each question from “none” to “always.” The scale’s questions are allocated among several factors, and the original score of each factor is calculated. These original scores are then translated into standard *T* scores in accordance with the assessment system standards. The *T* score is a standardized score that converts the original score into a numerical value suitable for comparison across various sizes and populations. The mean of the *T* score is often set at 50, with a standard deviation of 10. The formula for calculating the *T* score is shown in Equation (1):


(1) $$T=50+10\times \frac{X-M}{SD}$$

where *X* denotes the individual’s original score, *M* signifies the sample mean, and SD indicates the sample standard deviation. Thus, the distribution of *T* values is in the theoretical range of around 20 to 80. The *T* score was used to standardize the measurements in order to better assess students’ mental health.

Mental health status can be categorized into three levels based on the range of *T* values. A *T* score of 66 or above shows evident symptoms; a *T* score of 43 ~ 65 indicates likely or occasional symptoms; and a *T* score of 42 or lower indicates no clear symptoms. The CCSMHS scale has great reliability in assessing the mental health of university students, with Cronbach’s *α* coefficients ranging from 0.733 to 0.855. The scale’s practical use can help to comprehend the mental health state of university students while also providing a scientific basis for targeted care. Table 2 shows the distribution of mental health scores among university students in the sample.

**Table 2:** Mental health status of the sample.

Variable	*T* score				
	Mean	Median	SD	Min	Max
Somatization	47.5	45	9.1	19	112
Anxiety	48.5	47	10.2	19	95
Depression	47.3	46	9.4	20	99
Inferiority complex	47.5	45	10.0	20	99
Social withdrawal	48.4	47	9.7	22	93
Social aggression	46.1	44	9.0	17	109
Psychosexual disorder	45.8	43	7.2	22	116
Paranoid	46.1	44	9.2	20	100
Compulsion	48.6	47	10.0	16	91
Dependency	48.9	48	9.9	19	92
Impulsivity	47.0	45	8.9	17	111
Psychotic tendencies	47.4	44	9.1	15	113

SD refers to standard deviation.

This study analyzed academic performance using a comprehensive score and an English score to determine whether there is a difference in the influence of mental health on comprehensive academic performance and single-subject performance. Comprehensive academic performance is reflected by the comprehensive score, which is calculated as the weighted average of the student’s scores in each course, with each course’s weight set by the proportion of its credits relative to the total credits for that semester. In other words, it’s a combined score for all subjects. Single-subject performance is measured by an English score. As a general course, English is required for students in all majors, and it is selected as the research object for single-subject performance.

To examine the impact of mental health on short-term and long-term academic performance, this study uses the scores of the first examination (on December of the enrollment year) to represent the short-term impact and the scores of the second examination (on June of the second year of enrollment) to represent the long-term impact. The comprehensive and English scores of freshmen enrolled in 2016, 2017, and 2018 in the year of enrollment and the second year were matched with mental health census data, respectively. The data matching process rigorously followed the correspondence between the student ID and the semester to ensure data accuracy and consistency. Table 3 presents the comprehensive and English scores of freshmen at each enrollment year for further analysis.

**Table 3:** The academic performance of the sample.

Subject	Enrollment year	Semester	Mean	Median	SD	Min	Max
Comprehensive score	2016	1	68.8	70.0	12.0	10.0	96.1
		2	66.9	69.0	12.3	6.0	94.0
	2017	1	68.9	70.0	11.7	19.0	95.0
		2	67.4	68.7	11.3	0.0	96.0
	2018	1	75.8	77.4	10.0	31.0	96.6
		2	75.5	76.4	9.7	31.0	100.0
English score	2016	1	72.4	74.0	10.7	10.0	96.0
		2	69.4	70.0	9.7	6.0	94.0
	2017	1	69.2	70.0	11.0	19.0	94.0
		2	68.7	70.0	9.9	0.0	96.0
	2018	1	76.7	78.0	9.6	17.0	95.0
		2	76.0	77.0	9.4	31.0	100.0

SD refers to standard deviation.

### 2.2 Model

A multiple-regression model was employed to determine the impact of different mental health factors on academic performance. The dependent variable in the model was the students’ comprehensive or English score, while the independent variables were 12 standard scores of mental health factors (mental health variables) and two categorical variables: gender and enrollment year. The Variance Inflation Factor (VIF) was used to examine the collinearity of the 12 mental health variables, and the results showed that the VIF of these 12 variables was less than 5, indicating a low degree of collinearity between the variables. Multicollinearity in the model’s independent variables has no substantial effect on the robustness of the regression results.

Given the potential interaction between categorical and mental health variables, the fundamental structure of the multiple regression model developed in this paper is as shown Equation (2):


(2) $$\begin{array}{c} \mathrm{score}=a_{0}+a_{1}\mathrm{gender}+a_{2}EY+a_{3}\mathrm{gender}\cdot EY+\underset{i}{\sum }b_{1i}X_{i}\\ +\underset{i}{\sum }b_{2i}\mathrm{gender}\cdot X_{i}+\underset{i}{\sum }b_{3i}EY\cdot X_{i}+\underset{i}{\sum }b_{4i}\mathrm{gender}\cdot EY\cdot X_{i}+\varepsilon \end{array}$$

where score is the student’s comprehensive or English score, Xi is the mental health variable, gender is the gender categorical variable, and EY is the enrollment year categorical variable. Both gender and year of enrollment were regarded as dummy variables, with women and the year of 2016 serving as the control group (value 0), to investigate the effect of gender or enrollment year on academic performance. The model also analyzed how gender, enrollment year, and their interaction (gender∙EY∙Xi) affect students’ academic performance. *ε* represents the random disturbance term.

The model includes a total of 41 variables and interaction terms. If all variables are included in the regression equation, the model’s complexity and redundancy may grow. Bidirectional stepwise regression was utilized to simplify the model while also ensuring the reliability and interpretability of the regression results. Finally, the model will keep variables that have a significant impact on academic performance, providing a solid foundation for examining the impact of mental health problems on academic performance.

## 3 Results

The regression results are shown in Tables 4, 5. Each table displays unstandardized coefficients and standardized coefficients (Std.Beta) to highlight the effect size of each variable. The regression equation fits well in all four scenarios, and the results are all very significant. The model explains 14.1% of the variance in short-term comprehensive performance, 17.5% in long-term comprehensive performance, 16.5% in short-term English performance, and 16.6% in long-term English performance.

**Table 4:** The impact of gender, enrollment year, and mental health status on comprehensive score.

Variable	Short-term		Long-term	
	Coefficient	Std.Beta	Coefficient	Std.Beta
Intercept	73.535*** (1.337)	NA	73.884*** (1.281)	
Male	−0.706 (1.419)	−0.030	−4.225** (1.468)	−0.176
RY2017	−0.134 (1.660)	−0.006	−0.662 (1.606)	−0.027
RY2018	4.801* (1.974)	0.161	6.585*** (1.921)	0.220
Male∙RY2017	0.638 (0.519)	0.021	—	—
Male∙RY2018	2.901*** (0.645)	0.065	—	—
Somatization	−0.092*** (0.018)	−0.071	−0.120*** (0.021)	−0.092
Male somatization	—	—	0.092** (0.034)	0.180
Anxiety	0.131*** (0.023)	0.113	0.101*** (0.021)	0.086
Depression	−0.192*** (0.022)	−0.153	−0.183** (0.030)	−0.145
Male∙depression	—	—	−0.093** (0.033)	−0.187
RY2017 Depression	—	—	0.056 (0.034)	0.112
RY2018 Depression	—	—	0.122** (0.041)	0.199
RY2017∙Inferiority complex	—	—	—	—
RY2018∙Inferiority complex	—	—	—	—
Social withdrawal	−0.006 (0.023)	−0.005	—	—
RY2017∙Social withdrawal	0.071* (0.029)	0.145	—	—
RY2018∙Social withdrawal	0.095** (0.035)	0.160	—	—
Social aggression	0.106*** (0.022)	0.080	0.111*** (0.020)	0.084
Male∙social aggression	—	—	—	—
Psychosexual disorder	—	—	—	—
Paranoid	0.056* (0.023)	0.043	—	—
RY2017∙Paranoid	—	—	—	—
RY2018∙Paranoid	—	—	—	—
Compulsion	0.066*** (0.019)	0.056	0.054* (0.023)	0.045
Male∙compulsion	−0.045 (0.026)	−0.094	—	—
RY2017∙compulsion	—	—	0.054 (0.031)	0.111
RY2018∙Compulsion	—	—	−0.054 (0.037)	−0.092
Impulsivity	−0.041 (0.028)	−0.031	−0.048 (0.029)	−0.035
Male∙impulsivity	−0.084** (0.030)	−0.170	−0.056 (0.031)	−0.112
RY2017∙impulsivity	−0.071* (0.031)	−0.141	−0.084* (0.035)	−0.166
RY2018∙impulsivity	−0.082* (0.037)	−0.134	−0.032 (0.042)	−0.052
Psychotic tendencies	−0.066** (0.021)	−0.051	—	—
*p*-value	0.000	NA	0.000	—
*R* ^2^	0.141	NA	0.175	—

Standard errors in parentheses, ***p < 0.001, Extremely significant, **p < 0.01, Highly significant, *p < 0.05 Significant.

“RY” stands for “Enrollment Year,” e.g., “RY2017” stands for “enrollment year in 2017.” “Std.Beta” refers to standardized regression coefficient.

**Table 5:** The impact of gender, enrollment year, and mental health status on English score.

Variable	Short-term		Long-term	
	Coefficient	Std.Beta	Coefficient	Std.Beta
Intercept	77.720*** (1.188)	NA	73.034*** (1.045)	NA
Male	−4.324*** (1.136)	−0.196	−3.854*** (1.048)	−0.189
RY2017	−3.222* (1.384)	−0.143	−1.438 (1.335)	−0.069
RY2018	5.417*** (1.642)	0.197	7.404*** (1.595)	0.291
Male∙RY2017	0.935* (0.472)	0.033	0.904* (0.436)	0.034
Male∙RY2018	2.112*** (0.587)	0.051	−0.540 (0.543)	−0.014
Somatization	−0.109*** (0.016)	−0.091	−0.072*** (0.015)	−0.065
Male∙somatization	—	—	—	—
Anxiety	0.111*** (0.020)	0.104	0.055** (0.018)	0.056
Depression	−0.078*** (0.022)	−0.067	−0.105*** (0.018)	−0.098
Male∙depression	—	—	—	—
RY2017∙depression	—	—	—	—
RY2018∙depression	—	—	—	—
RY2017∙inferiority complex	−0.106** (0.036)	−0.233	—	—
RY2018∙inferiority complex	−0.010 (0.044)	−0.018	—	—
Social withdrawal	—	—	—	—
RY2017∙Social withdrawal	—	—	—	—
RY2018∙Social withdrawal	—	—	—	—
Social aggression	0.102*** (0.023)	0.083	0.080*** (0.021)	0.071
Male∙social aggression	−0.066** (0.024)	−0.142	−0.048* (0.022)	−0.111
Psychosexual disorder	−0.026 (0.0174)	−0.017	—	—
Paranoid	0.016 (0.031)	0.013	0.034 (0.018)	0.031
RY2017∙Paranoid	0.098* (0.040)	0.207	—	—
RY2018∙Paranoid	0.053 (0.048)	0.091	—	—
Compulsion	0.087*** (0.021)	0.079	0.034 (0.019)	0.034
Male∙compulsion	—	—	—	—
RY2017∙Compulsion	0.007 (0.029)	0.015	0.083*** (0.025)	0.201
RY2018∙Compulsion	−0.087* (0.036)	−0.162	−0.018 (0.030)	−0.036
Impulsivity	−0.091*** (0.017)	−0.074	−0.049* (0.022)	−0.043
Male∙impulsivity	—	—	—	—
RY2017∙Impulsivity	—	—	−0.074** (0.028)	−0.173
RY2018∙Impulsivity	—	—	0.000 (0.033)	0.000
Psychotic tendencies	−0.035 (0.019)	−0.029	—	—
*p*-value	0.000	NA	0.000	NA
*R* ^2^	0.166	NA	0.166	NA

Standard errors in parentheses, ***p < 0.001, Extremely significant, **p < 0.01, Highly significant, *p < 0.05 Significant.

“RY” stands for “Enrollment Year,” e.g., “RY2017” stands for “enrollment year in 2017.” “Std.Beta” refers to standardized regression coefficient.

### 3.1 Effect of gender and enrollment year on academic performance

The results imply that gender and enrollment year have significant effects on academic performance, particularly in the long run. As shown by the constant items, for female students enrolled in 2016 who did not have any mental health difficulties, the short- and long-term average scores were 73.54 and 73.88, respectively, while the short- and long-term average English scores were 77.72 and 73.03. In terms of gender influence, aside from the short-term impact on comprehensive scores, which was not significant—indicating no significant difference between the scores of male and female students who enrolled in 2016—all other scenarios showed a significant or highly significant gender effect. Specifically, the long-term average comprehensive scores of male students were 4.23 points lower than those of female students, with a Std.Beta (effect size) of −0.176, indicating a moderate negative impact of being male on long-term academic performance. For English scores, male students’ short-term scores were 4.32 points lower, with an effect size of −0.196, and their long-term scores were 3.85 points lower, with an effect size of −0.189. These standardized betas show a medium to strong effect size, suggesting that, after controlling for other variables, male students generally underperform academically.

The influence of the enrollment year on academic performance also differed significantly. Female students enrolled in 2017 had a considerably lower short-term average of English scores than those enrolled in 2016, with a difference of 3.22 points, and a standardized beta (effect size) of −0.143, indicating a modest negative effect. Meanwhile, there was no significant change in the short- and long-term average of comprehensive scores from 2016. In contrast, female students enrolled in 2018 had significantly higher long- and short-term average scores in both comprehensive and English than female students enrolled in 2016, with comprehensive short- and long-term average scores of 4.80 and 6.59 points higher, with effect sizes of 0.161 and 0.220, suggesting moderate to strong positive effects on academic performance. Additionally, their English scores of 5.42 and 7.40 points higher, with effect sizes of 0.197 and 0.291, indicating moderate to strong improvements. This implies a considerable improvement in academic performance among female students enrolled in 2018.

There was no significant difference in comprehensive scores between male students who enrolled in 2017 and 2016; however, the short-term average score in English was 0.94 points higher, with a standardized beta (effect size) of 0.033, suggesting a small positive effect. The long-term average score was 0.90 points higher, with a similarly modest effect size of 0.034. Male students enrolled in 2018 had considerably higher short-term average scores in comprehensive and English than those enrolled in 2016, by 2.90 and 2.11 points, respectively. The effect sizes were 0.065 for comprehensive scores and 0.051 for English scores, indicating small to moderate positive effects on short-term academic performance. However, there was no significant difference in long-term scores from 2016. This may represent changes in some academic and environmental aspects over time, influencing students’ short-term academic performance.

The results above show that the impacts of gender and enrollment year on academic performance are complicated and multifaceted, with not just gender differences considerably affecting academic performance but also significant fluctuations in academic performance across students enrolled in different years.

### 3.2 The impact of mental health status on academic performance

#### 3.2.1 Somatization

The results demonstrate that somatization had an extremely significant short- and long-term impact on comprehensive and English scores. Gender disparities were seen solely in the long-term impacts on comprehensive performance. Specifically, with every 1-point increase in the somatization score, the short-term average score fell by 0.09 points, with an effect size (Std.Beta) of −0.071, suggesting a modest but consistent negative impact. In the long term, this effect was amplified for female students, where each 1-point increase in somatization corresponded to a 0.12-point reduction (effect size: −0.092), compared to a smaller 0.03-point reduction for male students (effect size: −0.018). This difference in effect sizes underscores a gender disparity in the long-term academic impacts of somatization symptoms.

For English scores, each 1-point increase in somatization was associated with a 0.11-point reduction in short-term scores (effect size: −0.091) and a 0.07-point reduction in long-term scores (effect size: −0.065). These values indicate that somatization has a moderate and enduring negative impact on English performance, with relatively consistent effects over time.

This consequence indicates that the harmful impact of somatization symptoms on academic performance does not diminish significantly over time. Somatization is frequently connected with physical and emotional discomfort, including physical symptoms like headaches and exhaustion, which can be especially noticeable when students adjust to university life. Freshmen may be more susceptible to physical discomfort since they are in the early stages of adjusting to a new environment and role, which will impair their learning ability and academic performance. While students may eventually adjust, these physical symptoms may continue to have an impact on their academic performance.

#### 3.2.2 Anxiety

Anxiety had a strong short-term and long-term impact on comprehensive and English scores, but there was no significant difference based on gender or enrollment year. Specifically, for every 1-point increase in anxiety scores, the short-term average score of comprehensive performance increased by 0.13 points, with an effect size (Std.Beta) of 0.113, indicating a moderately positive effect on short-term academic performance. In the long term, each 1-point increase in anxiety was associated with a 0.10-point increase in comprehensive scores, with an effect size of 0.086, suggesting a consistent but slightly weaker positive impact over time.

For English scores, the short-term average rose by 0.11 points for each 1-point increase in anxiety (effect size: 0.104), showing a moderate effect. The long-term average English score increased by 0.06 points (effect size: 0.056), indicating a smaller, yet still positive, effect on English performance.

This positive correlation may indicate that mild anxiety improves academic performance. For university freshmen, anxiety at an early stage may originate from anxieties about their new environment, study tasks, and adjusting to university life, and this fear may motivate them to study harder. As the semester proceeds, the source of concern may gradually shift to demands connected to academic performance, scholarships, job prospects, and so on, which may continue to motivate students to work hard to some degree.

However, the impact of anxiety on academic performance differs based on the exact situation and intensity. In certain situations, especially when the task involves a threat-related stimulus, anxiety can enhance cognitive performance (16). For example, anxiety can strengthen the stimulus-driven attention system, resulting in improved performance. However, when anxiety becomes extreme or persistent (such as during exams), the effect becomes more complex.

Meanwhile, Karjanto and Yong (17) discovered that senior students may already have greater experience dealing with math test anxiety and are more likely to self-identify and seek assistance. Junior students, on the other hand, may not have abilities to control their anxiety, resulting in increased levels of anxiety during exams. Because such anxiety may hinder cognitive processes, making it more difficult for students to acquire knowledge effectively. Therefore, anxiety level and individual coping strategies play a vital role in this process.

In summary, moderate anxiety can increase students’ learning motivation; however, extreme anxiety can impair cognitive function and academic achievement.

#### 3.2.3 Depression

The depression score had a significant impact on the long- and short-term mean scores for both comprehensive and English scores. For every 1-point increase in depression ratings, the short-term mean score for comprehensive performance fell by 0.19 points, with an effect size (Std.Beta) of −0.153, indicating a strong negative impact on short-term academic performance.

The long-term impacts differed depending on gender and year of enrollment, with female students losing 0.18 points in comprehensive performance (effect size: −0.145, reflecting a moderate negative effect) and male students losing 0.28 points (effect size: −0.187, suggesting a stronger negative effect for males). This fact indicates that male students are more adversely affected by depression in their long-term comprehensive performance than female students.

The effect of depression on long-term comprehensive performance was not significantly different between 2017 and 2016, but for 2018 students, the long-term mean score dropped by 0.06 points (effect size: 0.199, indicating a moderate negative impact specific to this cohort), indicating that students enrolled in 2018 are more vulnerable to the long-term effects of depression on their academic performance compared to other cohorts. For English scores, for every 1-point increase in depression ratings, short- and long-term mean scores fell by 0.08 (effect size: −0.067, a modest negative effect) and 0.11 points (effect size: −0.098, indicating a moderate negative impact over time), respectively.

This occurrence suggests a permanent, unfavorable influence of melancholy mood on academic performance, which varies by gender and year of enrollment. Symptoms such as low mood, diminished motivation, and distraction often accompany depression, leading to reduced efficiency and accomplishment in academic activities, thereby affecting academic performance.

#### 3.2.4 Inferiority complex

The inferiority complex had a significant impact on the short-term English performance of students enrolled in 2017. For every 1-point increase in inferiority complex score, the mean English short-term score fell by 0.11 points, with an effect size (Std.Beta) of −0.233, indicating a strong negative impact on short-term English performance for this cohort. In other cases, the impact of the inferiority complex on academic performance was insignificant. Inferiority complexes may cause students to lose confidence and enthusiasm when faced with academic challenges, affecting their short-term academic performance. However, this influence may fade over time as students adjust to university life, as reflected by the lack of a significant long-term effect.

#### 3.2.5 Social withdrawal

The impact of social withdrawal on academic performance varies by year of enrollment. For every 1-point increase in the social withdrawal score, the short-term average score of enrolled students increased by 0.07 points in 2017 (effect size: Std.Beta = 0.145, indicating a moderate positive effect) and 0.10 points in 2018 (effect size: Std.Beta = 0.160, suggesting a slightly stronger positive effect). These effect sizes suggest that social withdrawal may have a modest yet beneficial impact on short-term academic performance for students who enrolled in these years.

However, after controlling for the year of enrollment, there was no significant impact of social withdrawal on academic achievement, suggesting that this effect is specific to certain enrollment years. This finding may indicate that, in some situations, socially disengaged students are more focused on academic work, reducing interference from external social activities with learning and boosting academic performance in the short term.

#### 3.2.6 Social aggression

Social aggression significantly influenced both long- and short-term mean comprehensive and English scores. Gender differences did not affect the influence of social aggression on comprehensive performance. For every 1-point increase in social aggression, the short-term and long-term averages increased by 0.11 points, with effect sizes (Std.Beta) of 0.080 and 0.084, respectively, indicating a moderate positive impact on academic performance over both timeframes.

Social aggression had varied effects on male and female students’ English scores. Female students’ short- and long-term average scores increased by 0.1 points, with effect sizes of 0.083 and 0.071, respectively, suggesting a consistent moderate positive effect. In contrast, male students’ scores increased by 0.04 and 0.03 points, with smaller effect sizes of −0.142 and − 0.111, respectively, indicating that social aggression may have a negative impact on male students’ English performance. These findings suggest that while social aggression positively influences female students’ academic outcomes, it may hinder male students’ English performance, potentially due to differences in how competitive or aggressive behaviors manifest in academic settings.

Social aggression may reflect a competitive or aggressive behavioral inclination that, in an academic situation, may translate into a strong pursuit of academic excellence, which in some students promotes better academic performance for some students, while for others, it may detract from academic focus.

#### 3.2.7 Paranoid

Paranoid had a significant impact on short-term comprehensive performance, which did not differ by gender or year of enrollment. Every 1-point rise in the paranoid score resulted in a 0.06-point increase in the short-term average comprehensive score, with an effect size (Std.Beta) of 0.043, indicating a small positive impact on short-term comprehensive performance. Furthermore, paranoid only had a significant impact on the short-term English scores of students enrolled in 2017, with every 1-point increase resulting in a 0.10-point increase in the short-term average English score (effect size: 0.207, suggesting a moderate positive effect specific to this cohort). Paranoid tendencies may lead students to be overly cautious and doubtful about their academic tasks, thereby increasing their academic performance in the near term as they invest additional time and attention. However, this performance may not be sustained long-term, as excessive caution and doubt could eventually hinder adaptability and academic confidence.

#### 3.2.8 Compulsion

Compulsive symptoms had a considerable favorable impact on both short- and long-term comprehensive performance. For every 1-point increase in the compulsion score, the comprehensive short-term average score increased by 0.07 points, with an effect size (Std.Beta) of 0.056, indicating a small to moderate positive effect. In the long-term average score increased by 0.05 points, with an effect size of 0.045, suggesting a modest positive impact on sustained academic performance.

The impact of compulsion on short-term English performance varies by year of enrollment. For students enrolled in 2016, every 1-point rise in compulsion score increases the short-term average English score by 0.09 points, with an effect size of 0.079, indicating a moderate positive effect. For students enrolled in 2018, compulsion had no meaningful influence on short-term English scores, suggesting that this positive effect is specific to earlier cohorts.

Furthermore, compulsion only had a significant influence on the long-term English scores of students enrolled in 2017, with the average long-term English score improving by 0.08 points for every 1-point rise, with an effect size of 0.201, indicating a strong positive effect specific to this cohort. This suggests that compulsive tendencies may enhance academic outcomes over a longer duration for 2017 students.

Students with compulsive symptoms may exhibit high focus and perfectionist inclinations in their academic assignments, resulting in improved short-term academic success. However, mental fatigue may reduce this effect over time, particularly if such tendencies are not guided effectively.

#### 3.2.9 Impulsivity

The impact of impulsivity on comprehensive scores differed by gender and year of enrollment. For every 1-point rise in impulsivity, male students’ short-term comprehensive performance declined by 0.08, with an effect size (Std.Beta) of −0.170, indicating a moderate negative impact specific to male students. Short-term comprehensive scores for students enrolled in 2017 and 2018 decreased by 0.07 and 0.08 points, respectively, with effect sizes of −0.141 and − 0.134, showing a moderate negative effect on short-term academic performance for these cohorts. These findings suggest that impulsivity negatively impacts students’ immediate academic outcomes, especially among those in these enrollment years.

Furthermore, impulsivity only had a significant effect on the long-term comprehensive scores of students enrolled in 2017, with each 1-point rise in the impulsivity score resulting in a 0.08-point decrease in the long-term comprehensive score, with an effect size of −0.166, indicating a sustained moderate negative impact for this group. Impulsivity had only a significant impact on the long-term performance of English students enrolled in 2017, with every 1-point rise in impulsivity scores resulting in a 0.07-point decrease, with an effect size of −0.173, suggesting a strong negative effect on English performance over time for this cohort.

Students with impulsive personalities may exhibit reduced focus and high-risk decision-making behavior during academic assignments, resulting in poor short-term academic performance. In the long run, this effect may be lessened if students strengthen their self-management abilities, potentially mitigating the negative impact of impulsivity on academic outcomes over time.

#### 3.2.10 Psychotic tendencies

Psychotic tendencies had a significant impact on comprehensive short-term performance. For every 1-point increase in the psychotic tendency score, the comprehensive short-term score decreased by 0.07 points, with an effect size (Std.Beta) of −0.051, indicating a modest negative effect on short-term academic performance. However, the impact on the long-term comprehensive score and English score was not significant. This finding implies that while psychotic tendencies may cause students to struggle academically in the short term, this effect may be mitigated over time through adaptation or intervention.

## 4 Conclusion

This study explored the influence of university freshmen’s mental health state on their short- and long-term comprehensive and single-subject academic performance, and the findings suggest that mental health factors may be classified into three categories:

### 4.1 Common factors

These factors have a significant impact on both comprehensive and single-subject academic performance, as well as short- and long-term outcomes. Examples include somatization, anxiety, depression, social aggression, and compulsion. These effect sizes indicate that these factors consistently influence academic outcomes across multiple domains and timeframes, with moderate to strong effects.

### 4.2 Category-specific factors

The impacts of these mental factors vary with gender, year of enrollment, and specific subject. For instance, social aggression had a stronger positive impact on female students’ English performance than on male students, and impulsivity showed a significant negative effect for male students and students enrolled in specific years. These findings suggest that social aggression and impulsivity may have different impacts based on gender and enrollment year.

### 4.3 Temporal factors

It only has a noticeable effect on short- or long-term performance. For instance, psychotic tendencies had a significant impact on short-term comprehensive scores, although this influence gradually decreased. This demonstrates that certain mental health issues may have a temporary impact on academic performance.

Furthermore, it is worth noting that some mental health factors may fall into all three categories, implying that they have an impact on both comprehensive and single-subject academic performance, as well as short- and long-term performance, and that this impact varies by gender, year of enrollment, and subject. For example, depression displayed variable impacts by gender and by enrollment year, underscoring its complex role in academic performance.

To better encourage university students to complete their studies, it is vital to pay attention to the mental health state of freshmen when they first enroll. University enrollment is a vital era for youth development, particularly in the first academic year, when freshmen confront the most mental problems, which can have a substantial impact on their academic success. According to the effect sizes, mental health issues like somatization, anxiety, and depression have particularly strong impacts on freshmen’s academic performance in both short- and long-term measures. Students will experience a variety of mental health issues throughout the first semester, compromising their academic performance. Freshmen are prone to loss, resistance, bewilderment, and other emotions when confronted with a foreign environment and new life obstacles, and they may even consider giving up. The aggregation of these mental health issues may impair their academic achievement. As a result, university officials and parents should work together to help students make the successful transition from senior high school to university. Furthermore, university officials should strengthen freshmen orientation and pay attention to students’ emotional shifts through guidance and communication, as well as provide emotional support to help them cope with mental stress and navigate the adjustment phase more readily. This is not only crucial for the freshmen’s development, but it also lays the groundwork for their future academic success.

Second, consider the impact of gender disparities and enrollment year on students’ mental health. The findings revealed that the impacts of certain mental health issues on academic performance were exclusively observed among students of a given gender or year of enrollment. For example, impulsivity had a moderate to strong negative effect on male students’ comprehensive scores and a substantial effect on students enrolled in specific years. So, when dealing with students’ mental health problems, we should take into account the differences in psychological state between males and females and provide appropriate interventions. Furthermore, changes in the social environment may result in considerable variances in psychological characteristics between students in different years of enrollment, as well as distinct mental health problems. Therefore, when tackling the mental health problems of students enrolled in all years, this aspect should also be taken into full consideration and specific assistance methods created.

The sample for this study consisted of students from three consecutive enrollment years at M University in Fujian Province, China. Future research should increase the sample size to include a greater number of university students and track their academic performance and employment in higher grades to better understand the influence of mental health during the university and job-hunting periods. Due to the scale’s restricted coverage, some mental health factors may have been excluded from the analysis. Future studies can use qualitative comparative analysis (QCA) approaches, as well as a combination of quantitative and qualitative research methods, to provide a more in-depth and comprehensive examination of the relationship between university students’ mental health state and academic performance.

## Data Availability

The data analyzed in this study is subject to the following licenses/restrictions: the dataset may only be accessed by authorized individuals within the Univerisity mentioned in the manuscript. External sharing or distribution is not permitted without prior approval. The dataset is provided strictly for academic or research purposes and cannot be used for commercial gain. The dataset contains sensitive personal information that must be anonymized before any analysis or publication. Identifiable information should be removed or encrypted to protect individuals’ privacy. Requests to access these datasets should be directed to Cheng Li, bigbosscool1990@live.com.

## Footnotes

^1^Somatization is the term for a person who has emotional or psychological problems but does not show psychological signs and then manifests numerous physical symptoms. In other words, when an individual has anxiety, depression, tension, or other emotional distress, they experience true physical discomfort or pain, which is a manifestation of the transition of psychological distress into physical symptoms.
