# Supplementary material for: The impact of university freshmen’s mental health on academic performance: an empirical study based on M University in Fujian Province, China
Source: Front Public Health. 2024 Dec 13;12:1483031. doi: 10.3389/fpubh.2024.1483031 (PMC11671369; doi:10.3389/fpubh.2024.1483031)
Supplement: Supplementary file 1 [file Data_Sheet_1.pdf]

Supplementary Material

中国大学生心理健康量表

指导语：以下列出了一些人可能会有的问题，请仔细阅读每一条，然后根据自己最近一个月的实际感觉，选择最符合您的一种情况，并在答题卡的相应位置上用 2B 铅笔涂好。其中“没有”涂 1，“偶尔”涂 2，“有时”涂 3，“经常”涂 4，“总是”涂 5。

题号	题目	没有 1	偶尔 2	有时 3	经常 4	总是 5
1	头痛或头晕					
2	浑身软弱无力					
3	做事情必须反复检查					
4	不能容忍自己的缺点					
5	为做事达不到预定目标而烦恼					
6	不允许出现差错					
7	无法控制自己重复做某些事，如：不停地检查门是否关好，不断地洗手或数数等					
8	头脑中有不必要的想法或字词盘旋					
9	感到手或脚沉重					
10	有困难时没人帮助，会无所适从					
11	做事不主动					
12	担心朋友会不理我					
13	不得不独立做某事的时候，仍希望有人帮助					
14	觉得很多事情自己处理不好					
15	没有别人安排，不知道该做什么					
16	如果老师不布置任务，便不知道该干什么					
17	怕承担事情的后果					
18	明知是小事，还是会发火					
19	想占便宜					
20	对讨厌的人进行直接攻击					
21	突发奇想地行事					
22	忍不住伤害令自己不满的人					
23	不顾场合大发雷霆					
24	做事情不考虑后果					
25	莫名其妙地对别人发火					
26	怀疑自己有不治之症					
27	感到很少有人理解自己					
28	对性的向往有罪恶感					
29	想骂人					
30	听到旁人听不到的声音或看到旁人不能看到的					

	东西					
31	手淫后有悔恨感和负罪感					
32	怀疑旁人知道我内心的想法					
33	憎恨周围的人					
34	容易疲乏					
35	感到烦闷、坐立不安					
36	过于敏感					
37	容易紧张					
38	失眠					
39	想到坏得说不出口的事					
40	对将来感到恐惧					
41	感到任何事情都很困难					
42	对一些事情感到提心吊胆					
43	对他人大发脾气					
44	对学习没有兴趣					
45	想偷懒					
46	难以集中精力做事					
47	胸闷					
48	感到生活空虚、无趣					
49	想占便宜					
50	感到自己没有价值					
51	处于高度警觉状态					
52	觉得自己缺乏吸引力					
53	猜疑别人会伤害我					
54	觉得自己是一个失败者					
55	恶心					
56	担心别人瞧不起自己					
57	自己的才能得不到发挥					
58	觉得自己总是给他人带来麻烦					
59	没有人真心关心我					
60	觉得自己无论怎么努力都不会成功					
61	怕别人注意					
62	想骂人					
63	同异性相处时感到害羞					
64	参加聚会感到焦虑					
65	与权威人士谈话感到紧张					
66	有想摔打或破坏东西的冲动					
67	参加小组讨论感到紧张					
68	与陌生人见面就脸红					
69	呼吸有困难					
70	与他人争论不休					
71	沮丧、郁闷					
72	有想打人或伤害他人的冲动					

73	在人群中感到不自在					
74	容易激动					
75	当别人取得成绩时，很想打击他(她)一下					
76	嫉妒比自己幸运的人					
77	觉得别人爱出风头					
78	感到一阵阵发冷或发热					
79	觉得性行为是可耻的					
80	想到坏得说不出口的事					
81	对异性的好奇使我有罪恶感					
82	感到有人在监视自己，谈论自己					
83	觉得性梦是下流的					
84	感到别人能控制自己思想					
85	想偷懒					
86	担心自己处女膜破损或性无能					
87	感到别人对自己怀有敌意					
88	怀疑自己的能力					
89	感到没有可以信任的人					
90	别人对我的侮辱让我耿耿于怀					
91	感到别人的善意都是有目的的					
92	别人对我的成绩没有做出恰当的评价					
93	过度关注别人对我的反应					
94	不愿听从别人的意见					
95	生活习惯被打乱，会很生气					
96	做事情必须做得很慢以保证做得正确					
97	性幻想使我不能集中精力学习					
98	感到前途没有希望					
99	对看不惯的人或事，会忍不住去指责					
100	感到压抑					
101	害怕当众说话					
102	对一些无关紧要的事情感到过分忧虑					
103	感到有人要迫害自己					
104	觉得遗精(或月经)是肮脏的					

Chinese College Students Mental Health Scale (English version)

Instructions: The following are some issues that individuals may experience. Please read each item carefully and choose the option that best describes your feelings in the past month. Mark your answer on the answer sheet using a 2B pencil. Mark "1" for "None," "2" for "Occasionally," "3" for "Sometimes," "4" for "Often," and "5" for "Always."

Item No.	Question	None 1	Occasionally 2	Sometimes 3	Often 4	Always 5
1	Headaches or dizziness					
2	Feeling weak all over					
3	Must repeatedly check things					
4	Cannot tolerate my shortcomings					
5	Worry about not achieving goals					
6	Cannot allow for mistakes					
7	Uncontrollably repeat actions, like checking if the door is locked, washing hands repeatedly					
8	Unnecessary thoughts or words circling in my mind					
9	Feel heaviness in hands or feet					
10	Feel helpless when no one helps during difficulties					
11	Not proactive in doing things					
12	Worry friends will ignore me					
13	Even when having to do something independently, wish someone would help					
14	Feel that I cannot handle many things well					
15	Don't know what to do without others' arrangements					
16	If the teacher doesn't assign tasks, don't know what to do					
17	Fear of bearing the consequences					
18	Get angry even over small things					
19	Want to take advantage of others					
20	Directly attack people I dislike					

21	Act on sudden impulses					
22	Can't help hurting people who displease me					
23	Lose my temper regardless of the situation					
24	Do things without considering the consequences					
25	Get angry at others for no reason					
26	Suspect I have an incurable disease					
27	Feel that few people understand me					
28	Feel guilty for sexual thoughts					
29	Want to curse others					
30	Hear or see things others cannot					
31	Feel guilt and remorse after masturbation					
32	Suspect others know my inner thoughts					
33	Hate the people around me					
34	Get tired easily					
35	Feel restless and uneasy					
36	Overly sensitive					
37	Easily get nervous					
38	Insomnia					
39	Think about things too terrible to mention					
40	Fear the future					
41	Feel everything is difficult					
42	Feel anxious about certain things					
43	Lose my temper at others					
44	Have no interest in studying					
45	Want to be lazy					
46	Difficulty concentrating					
47	Chest tightness					
48	Feel life is empty and meaningless					
49	Want to take advantage of others					
50	Feel I am worthless					
51	In a state of high alert					

52	Feel unattractive					
53	Suspect others will harm me					
54	Feel like a failure					
55	Nausea					
56	Worry that others look down on me					
57	Feel my talents are not fully utilized					
58	Feel like I am always troubling others					
59	Feel no one genuinely cares about me					
60	Feel like no matter how hard I try, I will not succeed					
61	Fear others' attention					
62	Want to curse others					
63	Feel shy around the opposite sex					
64	Feel anxious at social gatherings					
65	Feel nervous talking to authority figures					
66	Impulse to break or destroy things					
67	Feel nervous during group discussions					
68	Blush when meeting strangers					
69	Difficulty breathing					
70	Argue with others incessantly					
71	Feel depressed					
72	Impulse to hit or hurt others					
73	Feel uncomfortable in crowds					
74	Easily excited					
75	Feel the urge to undermine others' success					
76	Jealous of those more fortunate than myself					
77	Feel others like to show off					
78	Feel sudden chills or heat					
79	Feel that sexual behavior is shameful					
80	Think about things too terrible to mention					

81	Feel guilty for curiosity about the opposite sex					
82	Feel someone is watching or talking about me					
83	Feel that sexual dreams are obscene					
84	Feel others can control my thoughts					
85	Want to be lazy					
86	Worry about damage to my hymen or sexual impotence					
87	Feel others are hostile towards me					
88	Doubt my own abilities					
89	Feel there is no one I can trust					
90	Resent insults from others					
91	Feel others' kindness has ulterior motives					
92	Feel my achievements are not properly recognized					
93	Overly concerned with others' reactions to me					
94	Unwilling to listen to others' opinions					
95	Get angry when my daily routine is disrupted					
96	Must do things very slowly to ensure they are done correctly					
97	Sexual fantasies prevent me from concentrating on studies					
98	Feel hopeless about the future					
99	Can't help but criticize people or things I dislike					
100	Feel oppressed					
101	Fear public speaking					
102	Overly worried about trivial matters					
103	Feel someone is trying to persecute me					
104	Feel that wet dreams (or menstruation) are dirty					
